# Middle Meningeal Artery Embolization for the Management of Chronic Subdural Hematoma

**DOI:** 10.7759/cureus.47293

**Published:** 2023-10-18

**Authors:** Balakrishna Aggipothu, Saikanth Deepalam, Sagar Badachi, Prabakaran Palanisamy, Sharath Kumar GG, Harshith Kramadhari, Gurtej singh Sardar, Thomas Mathew, Ragunandan Nadig, GRK Sarma

**Affiliations:** 1 Intervention Neuroradiology, St John's Medical College and Hospital, Bengaluru, IND; 2 Intervention Neuroradiology, St. John's Medical College and Hospital, Bengaluru, IND; 3 Neurology, St John's Medical College and Hospital, Bengaluru, IND; 4 Interventional Neuroradiology, St John's Medical College and Hospital, Bengaluru, IND; 5 Intervention Neuroradiology, St John's Medical College and Hospital, Bangalore, IND; 6 Neurology, St John's Medical College and Hospital, Bangalore, IND

**Keywords:** embospheres, antiplatelet subdural hematoma, mma embolization, middle meningeal artery (mma), chronic sdh

## Abstract

Introduction

Chronic subdural hematoma (cSDH) results from neovascularization of the subdural capsular membrane and embolization of the Middle Meningeal Artery (MMA) helps inhibit the same and prevent recurrence.

Materials and methods

We retrospectively reviewed the endovascular management for chronic SDH in 29 patients between 2018 to 2022. The parameters analyzed were clinical history, radiologic imaging findings, procedure details, and angiographic and clinical outcomes.

Results

Twenty-nine MMA embolization procedures were done. Follow-up MRI or CT done in 28 subjects, showed complete resolution in 25 patients and a significant reduction in bilateral SDH in three patients. One patient died due to renal failure and aspiration pneumonia-related complications. Ninety days mRS (modified Rankin scale) was 0 in 25 patients (86%), one in two patients, and two in one patient.

Conclusions

MMA embolization for chronic SDH is a feasible, safe, and effective technique in patients with chronic and recurrent SDH.

## Introduction

Chronic subdural hematoma (cSDH) is the subdural collection of blood over a period of three weeks. The presentation of patients with cSDH is usually with behavioral and progressive reversible cognitive decline, chronic headache, gait instability, and impairment of speech. Patients also present with seizures, focal neurological deficits, and hemiparesis. With increasing age and the common use of antiplatelets and anticoagulation medication, the incidence of SDH has increased over the years, with Western data showing rates almost double the rate of subarachnoid hemorrhage from aneurysms [1].

Surgical options available for management include burr-hole, twist drill, or craniotomy with or without drain placement. The recurrence rates after surgical evacuation are 5-30% [2]. The risk factors for recurrence are antiplatelet/anticoagulation therapy, post-operative residual air in subdural space, liver dysfunction, and diabetes. The recurrence is a result of inflammation and neovascularization. Fibrinolysis with the liquefaction of the initial subdural clot stimulates inflammation and dural thickening, inciting angiogenesis with the formation of immature capillaries. These capillaries have leaky vascular membranes and repeatedly lead to micro hemorrhages resulting in recurrence of the SDH.

The rate of physiological reabsorption is exceeded by repeated micro-hemorrhages due to which subdural hemorrhage progressively enlarges. Thus, the entire basis for the pathology is the formation of leaky vascular membranes, which incite a positive feedback cycle of continued hemorrhage, inflammation, and angiogenesis [3]. The treatment strategies aim to disrupt this cycle and shift the balance in favor of physiological reabsorption of the hemorrhage. Studies on leaky vascular membranes demonstrated that they are mostly supplied by the middle meningeal artery (MMA), hence MMA embolization in cSDH inhibits neovascularization of the outer membrane and prevents maintenance of the hematoma [4, 5]. Our study aims to describe the outcome of MMA embolization with embospheres in patients with chronic SDH under local anesthesia.

## Materials and methods

We report a single-center experience of outcomes of patients who underwent middle meningeal artery embolization for chronic SDH from 2018 to 2022 in our department by retrospective analysis. Patient details including risk factors, pre-operative radiological imaging features, endovascular procedure details, their associated complications, patient’s neurological status at the time of discharge, and post-procedural follow-up were noted. 

Imaging 

All the patients who are suspected and history of having SDH in the past, underwent appropriate imaging by either magnetic resonance imaging (MRI) or computed tomography (CT). Radiological features like chronic SDH or acute on chronic SDH causing midline or mass effect, thickened inner dural layer, and presence of membrane within SDH were noted.

Patient selection 

All patients who were at risk for surgery including old age more than 60 years, those who were not willing and fit for surgery, and patients with recurrent SDH after surgery were included in this study for the period from 2017 to 2022. Patients with significant midline shifts of more than 5 mm were not included in this study and needed emergency decompressive craniotomy/craniectomy.

Endovascular method

Middle meningeal artery embolization was done under local anesthesia for all patients. Arterial access via the femoral artery was maintained with a short sheath (6F). Cerebral angiograms were obtained with a 5F vertebral glide catheter. Confirmation of the ophthalmic artery and choroidal blush was done from selective internal carotid artery angiography.

Diagnostic cerebral angiograms performed from the bilateral external carotid artery (ECA) showed a hypertrophied MMA and blush overlying the cerebral convexities. The 5F vertebral catheter was exchanged with the 6F envoy catheter which was navigated up to the ostium of the right ECA and into the ECA distally. Using a microcatheter/microwire combination the MMA was cannulated and angiograms were obtained to confirm the position. The microcatheter tip was placed close to the MMA bifurcation away from the foramen spinosum.

**Figure 1:**
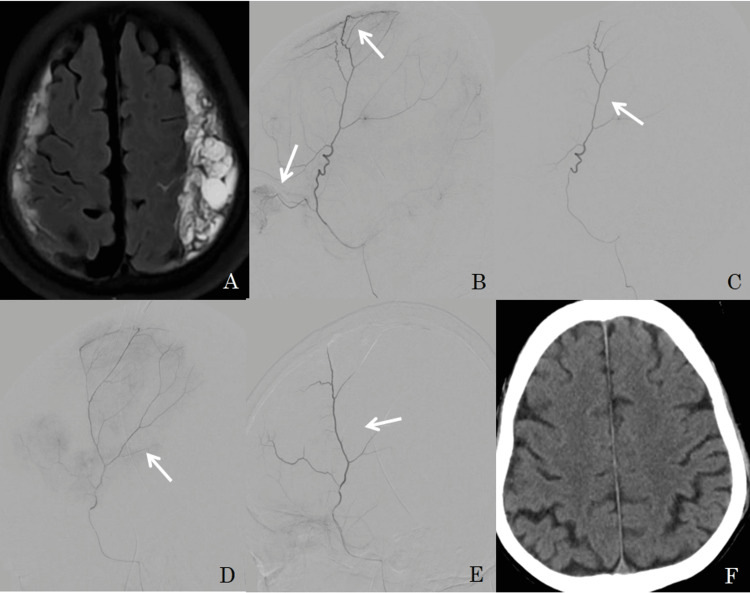
A 67-year female presented with bilateral SDH (image A), on selective catheterization of right MMA, shows tortuous frontal and parietal branches with abnormal blush (image B) and absent blush following embolization (image C) (arrow-head). Images D&E show pre and post-MMA embolization of left MMA, and complete resolution in a 6-month follow-up CT (image F).

**Figure 2:**
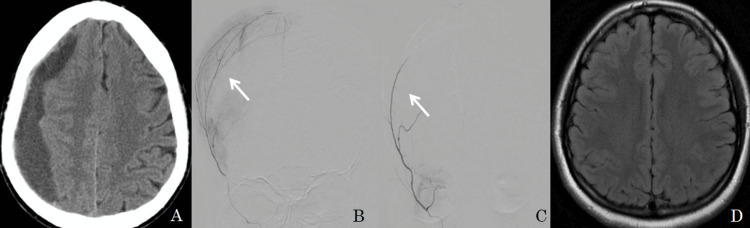
A 65-year-old male presented with right-side chronic SDH (image A). On selective catheterization of the right MMA shows tortuous frontal and parietal branches and abnormal blush (image B). Embolization with embospheres was performed and a post-embolization angiogram (image C) shows an absence of blush and a follow-up MRI after 6 months (image D) shows complete resolution.

Slow injection of 100-300 microns embospheres was performed under fluoroscopy till the MMA was occluded. Check angiogram revealed complete occlusion of the MMA. The femoral arterial sheath was removed and hemostasis was achieved by manual compression. Immediate DynaCT was done after the procedure and a follow-up CT/MRI scan after three and six-month intervals. Post embolization cone beat CT (Dyna CT) is done to see enhancement of the hematoma membrane, which indicates embospheres particles penetrate the hematoma membrane and MMA. Figure 1 shows bilateral MMA embolization in bilateral cSDH and Figure 2 shows unilateral MMA embolization in unilateral cSDH.

## Results

The retrospective study included 29 patients (10 female and 19 male) with ages ranging from 45 years to 90 years. Table 1 shows patient characteristics and clinical data.

**Table 1:** Patient characteristics and clinical data Y: years, M: male, F: female, mRs: modified Rankin scale, cSDH: chronic subdural hemorrhage, CT: computed tomography, MRI: magnetic resonance imaging.

Serial no	Age &Gender	Co-morbidities	Chronic SDH	Embolization material	90 days mRS	6 months follow-up MRI/CT	
1	63 Y & M	Diabetic, Hypertension	Bilateral	Embospheres	0	Significant reduction	
2	87 Y & M	Renal failure	Bilateral	Embospheres	6	-	
3	60 Y & F	Liver disease	Unilateral	Embospheres	0	Complete resolution	
4	65 Y & M	On Anticoagulation	Bilateral	Embospheres	1	Complete resolution	
5	72 Y & M	On Anticoagulation	Bilateral	Embospheres	2	Significant reduction	
6	55 Y &M	Hypertension, Diabetic	Bilateral	Embospheres	0	Complete resolution	
7	46Y & M	Budd chairi syndrome	Bilateral	Embospheres	1	Complete resolution	
8	74Y &M	On Anticoagulation	Unilateral	Embospheres	0	Complete resolution	
9	86Y & M	On Anticoagulation	Unilateral	Embospheres	0	Complete resolution	
10	75 Y & M	On Anticoagulation	Unilateral	Embospheres	0	Complete resolution	
11	55 Y & F	Diabetic, Hypertension	Unilateral	Embospheres	0	Complete resolution	
12	67Y & F	Diabetic, Hypertension	Bilateral	Embospheres	0	Complete resolution	
13	57 Y & F	On Anticoagulation	Unilateral	Embospheres	0	Complete resolution	
14	66 Y & M	Liver disease	Bilateral	Embospheres	0	Complete resolution	
15	78 Y & F	Diabetic, Hypertension	Unilateral	Embospheres	0	Complete resolution	
16	57 Y & M	Diabetic, Hypertension	Bilateral	Embospheres	0	Significant reduction	
17	72Y & F	On Anticoagulation	Unilateral	Embospheres	0	Complete resolution	
18	65 Y & M	Diabetic, Hypertension	Bilateral	Embospheres	0	Complete resolution	
19	66 Y & F	Diabetic, Hypertension	Unilateral	Embospheres	0	Complete resolution	
20	71Y& M	Hypertension	Bilateral	Embospheres	0	Complete resolution	
21	64Y & F	Hypertension	Unilateral	Embospheres	0	Complete resolution	
22	62Y & M	On Anticoagulation	Unilateral	Embospheres	0	Complete resolution	
23	63Y & F	Hypertension	Unilateral	Embospheres	0	Complete resolution	
24	54 Y & M	Diabetic	Bilateral	Embospheres	0		Complete resolution
25	57Y & F	On Anticoagulation	Bilateral	Embospheres	0		Complete resolution
26	56 Y &M	On Anticoagulation	Unilateral	Embospheres	0		Complete resolution
27	62Y &M	On Anticoagulation	Bilateral	Embospheres	0		Complete resolution
28	67Y&M	On Anticoagulation	Unilateral	Embospheres	0		Complete resolution
29	61 Y &M	On Anticoagulation	Unilateral	Embospheres	0		Complete resolution

The most common symptoms were headache, and cognitive decline (22/29; 75 %), followed by focal neurologic deficits (10/29; 34%), the focal neurological deficit with aspiration pneumonia (1/29; 0.03%), and seizures (7/29; 24%). Bilateral chronic SDH was seen in 14/29 and unilateral in 15/9 patients. Thirteen of 29 patients were taking anticoagulant medicine. MMA embolization was performed under local anesthesia for all 29 patients. Occlusion of the frontal and parietal branches of the MMA was confirmed.

On follow-up, twenty-eight patients had a good outcome with no recurrence of subdural hematoma. One patient died due to renal failure and aspiration pneumonia-related complications. 

The modified Rankin scale (mRS) was used to measure the degree of disability after MMA embolization. Ninety days mRS was 0 in six patients (86%), one in two patients, and two in another patient. Follow-up MRI/CT done in 28 subjects, showed complete resolution in 25 patients and significant reduction in SDH in three patients.

## Discussion

Acute SDH is the accumulation of venous blood in the subdural space due to a tear in the bridging veins [6]. Chronic SDH is due to injury to dural border cells which are capable of laying down fibro-cellular connective tissue. When dural border cells are injured, it causes an inflammatory reaction, leading to fibrogenesis and angiogenesis [7]. SDH in liver disease, renal failure, and budd chiari syndrome was spontaneous and secondary to thrombocytopenia which was managed with platelet transfusion before the procedure [8].

There are no specific guidelines for cSDH management. cSDH with minor symptoms, <10 mm in thickness, and with midline shift <5 mm are managed conservatively [9]. Surgical management such as craniotomy and burr-hole is considered in patients with severe symptoms or a larger volume of cSDH. There have been favorable outcomes after such surgical procedures; however, there are high chances for the SDH to recur. There is also the need for reversal of antiplatelet and anticoagulant agents prior to surgery. Thus, avoiding surgery is beneficial in some patients and less invasive options are attractive for selected populations, such as the elderly with major co-morbidities that could complicate surgery [10]. Ex-vivo studies of the hematoma capsule have shown that the outer membrane consists of leaky micro-capillaries which result in repeated micro-hemorrhages. These studies have also suggested that this outer membrane is supplied by the MMA and embolization of the MMA will inhibit angiogenesis and hence the cycle of neovascularization and inflammation which is responsible for the maintenance of the hematoma [4, 5].

The polyvinyl alcohol (PVA) particles penetrate the capsular membrane in addition to the MMA as indicated by the enhancement seen on DynaCT. This dissipation of contrast medium further confirms the leakiness of the capillaries in the outer capsular membrane. MMA embolization leads to occlusion of the source of leakage leading to disruption of the angiogenesis and inflammation cycle. Thus the balance shifts in favor of hematoma resorption [11]. In our study, we used embospheres which are better embolic agents than PVA which was used in previous studies. Embospheres are smooth and spherical in shape and cause less fragmentation, aggregation, and catheter occlusion compared to PVA [12].

In a study of selective angiography in patients with cSDH (n=35), Tanaka et al. found diffuse dilatation of the MMA and its branches forming an abnormal vascular network [5]. Hashimoto et al. described this pattern of immature neo-vessels as ‘cotton-wool-like staining’. This pattern is no longer observed after embolization indicating successful blockage. This forms the basis for MMA embolization [13]. Post-embolization non-contrast head CT may show the disappearance of this pattern, indicating successful blockage [14]. In a meta-analysis and systemic review by Srivatsa et al. in 2019, the recurrence rates of SDH in patients undergoing embolization were significantly lower (2.1%) as compared with the conventional treatment group (27.7%). The complication rates were similar and were 2.1% in the embolization group and 4.4% in the conventional treatment group. Modified Rankin scale score >2 in the embolization (12.5%) versus conventional treatment (9.1%) group also showed no statistical difference (P = 0.689) [15]. A comparative study consisting of 541 patients by Ban et al., compared MMA embolization and conservative management in cSDH which shows complete resolution in asymptomatic SDH and symptomatic patients as well except one patient. The treatment failure rate in the embolization group was lower than in the conventional treatment group (one of 72 patients [1.4%] vs 129 of 469 patients [27.5%] [16]. In another comparative study of MMA embolization vs. burr hole craniotomy, Kim et al. found that the rates of recurrence post-embolization were significantly lower (3.8%) than those who underwent craniotomy (33.3%). The rate of repeat craniotomy in the latter was 20.8%. The study thus concluded that the preferred treatment in recurrent cSDH should be MMA embolization [17].

Our study showed complete resolution in twenty-five patients without recurrence and a significant reduction in bilateral SDH in three patients without the need for surgery. One major limitation of this study is the retrospective, which is prone to selection biases. Another limitation of the study was a small sample size and long-term follow-up not available. There is a further need for more randomized control trials (RCTs) to conclude appropriate patient selection, optimal techniques of embolization, and embolization timing.

## Conclusions

MMA embolization for chronic SDH is feasible, safe, and an effective technique since surgical procedures for cSDH are associated with high recurrence and complication rates. Unlike previous researchers, we used Embospheres as an embolic agent for MMA embolization instead of PVA in cSDH under local anesthesia.
